# *bcRep*: R Package for Comprehensive Analysis of B Cell Receptor Repertoire Data

**DOI:** 10.1371/journal.pone.0161569

**Published:** 2016-08-23

**Authors:** Julia Bischof, Saleh M. Ibrahim

**Affiliations:** Institute of Experimental Dermatology, University of Lübeck, Lübeck, Germany; INRA, FRANCE

## Abstract

Immunoglobulins, as well as T cell receptors, play a key role in adaptive immune responses because of their ability to recognize antigens. Recent advances in next generation sequencing improved also the quality and quantity of individual B cell receptors repertoire sequencing. Unfortunately, appropriate software to exhaustively analyze repertoire data from NGS platforms without limitations of the number of sequences are lacking. Here we introduce a new R package, *bcRep*, which offers a platform for comprehensive analyses of B cell receptor repertoires, using IMGT/HighV-QUEST formatted data. Methods for gene usage statistics, clonotype classification, as well as diversity measures, are included. Furthermore, functions to filter datasets, to do summary statistics about mutations, as well as visualization methods, are available. To compare samples in respect of gene usage, diversity, amino acid proportions, similar sequences or clones, several functions including also distance measurements, as well as multidimensional scaling methods, are provided.

## Introduction

The immune system is a complex network of cells and organs that mainly defends the body against pathogens [1]. Lymphocytes, in particular B and T cells, are the major cellular components of the adaptive immune response. The highly diverse Immunoglobulins (IG) and T cell receptors (TR) provide specific immune reactions due to pathogen recognition.

Major advances in next generation sequencing (NGS) led to possibilities of deep sequencing of B and T cell receptor repertoires. Among others, immune repertoires of disease models [2, 3], as well as changes during aging [4] are of main interests.

Existing tools like IMGT/HighV-QUEST (tested version: 3.3.5; [5]) process raw IG/TR NGS data, while extracting V (variable), D (diversity) and J (joining) regions and defining special sequence parts like complementary determining regions (CDR) or framework regions (FR). However, to interpret these sequences and compare them among study groups, further analyses are required. Additionally, online tools for B and T cell repertoire analysis are available (e.g. Change-O, iRAP, IMEX, MiXCR or VDJtools [6–10]). Unfortunately, most of them are limited to either the number of input sequences or a limited number of analysis methods. Furthermore, the user is restricted to the output format generated by the program and individual output modifications are usually lacking. Whereas Change-O was designed to track somatic hypermutations of BCRs, iRAP was developed to characterize repertoire-level dynamics and diversity of B and T cell immune repertoires. IMEX analyzes diversity and clones of IGMT/HighV-QUEST data, while MiXCR concentrates on processing raw data to quantitated clonotypes. VDJtools can use several types of inputs, but also focusses mainly on clonotype data. Table 1 provides a comparison between *bcRep* and other selected IG analysis tools, like Change-O, iRAP and IMEX. *bcRep* comprises many functions in one package, where otherwise several tools are required.

**Table 1:** Comparison of the different B cell receptor repertoire analysis tools and *bcRep*.

feature	bcRep	Change-O	iRAP	IMEX
base	R package	command-line, R package	online tool	GUI, command line
input	IMGT/HighV-QUEST	IMGT/HighV-QUEST	FASTA	FASTA, IMGT/HighV-QUEST
special function to read input	+	+	-	-
combine several files	+	-	-	+
sequence number limited	-	-	+	-
comparison of samples	+	-	-	+
sequence filtering	+	-	-	-
sequence statistics	+	-	-	+
general mutation statistics	+	+	-	-
advanced mutation statistics	+	+	-	-
lineage trees	-	+	+	-
gene usage	+	-	+	+
gene/gene combinations	+	-	+	-
assemble clonotypes	+	+	+	+
clone filtering	+	-	-	-
clone statistics	+	-	+	+
shared clones	+	-	-	+
clone tracking	-	-	+	-
amino acid distribution	+	+	-	-
diversity	+	+	+	+
dissimilarities/distances on gene usage data	+	-	-	-
dissimilarities/distances on sequence data	+	+	-	-
multidimensional scaling	+	-	-	-
several visualization routines	+	-	+	+
alignment of sequences	-	+	-	-
estimation of repertoire size	-	-	+	-

‘+’ refers to feature exists,

‘-‘ refers to feature does not exist.

Information was taken from the documentation of the tools.

Here, we present a new R package [11], *bcRep*, for the analysis of IG repertoires. It comprises methods to combine and read IMGT/HighV-QUEST output files, and several methods to study not only clones, but also the total set of input sequences or subsets of sequences. Sequences can be filtered for their functionality or junction frame usage, and clones also for their size. Gene usage, as well as (silent and replacement) mutations and diversity can be analyzed. Clonotypes can be classified and compared between different samples. Several dissimilarity and distance measurements are available to analyze relations between gene usage or sequence data of different samples (beta diversity). Samples can not only be analyzed individually, but also compared to each other. Further it has no limitations regarding sequence numbers and is available for Unix, Mac OS X and Windows systems.

## Methods

In the following we describe data formats used as input and methods implemented in *bcRep*. An overview about all functions can be found in Table 2. The R package vignette provides a more detailed overview about the usage of functions and their outputs or visualization methods.

**Table 2:** Functions of the *bcRep* package and their description.

Function	Description
combineIMGT()	Combines several IMGT/HighV-QUEST outputs
readIMGT()	Reads IMGT/HighV-QUEST outputs and filters for sequences without results (optionally; see paragraph “Input data”)
sequences.functionality() sequences.junctionFrame()	Gives information about functionality and junction frame usage of input data
sequences.getAnyFunctionality() sequences.getProductives() sequences.getUnproductives()	Filters datasets for productive/unproductive sequences
sequences.getAnyJunctionFrame() sequences.getInFrames() sequences.getOutOfFrames()	Filters datasets for in-frame/out-of-frame sequences
sequences.mutation()	Summary statistics about mutations in V-region, FR1-3 or CDR1-2 sequences, like number of all mutations, number of silent/replacement mutations or R/S ratio
sequences.mutation.AA()	Analyzes all replacement mutations and returns a matrix with proportions of mutations from (germline) amino acid to mutated amino acid + visualization method
plotSequenesMutationAA()	
sequences.mutation.base()	Analyzes nucleotide distributions next to silent mutations (positions -3 to +3) + visualization method
plotSequencesMutationBase()	
clones()	Combines sequences to clonotypes with same V gene and J gene (optional) and a variable CDR3 sequence identity
clones.filterSize() clones.filter Functionality() clones.filterJunctionFrame()	Filters clones for their size, functionality or junction frame usage
clones.CDR3Length() plotClonesCDR3Length() plotClonesCopyNumber()	Statistics and visualizations of CDR3 length distribution and copy number of clones
clones.giniIndex()	Gini index of a set of clones
clones.shared() clones.shared.summary()	Clones shared between at least two samples. Same criteria than in clones()
geneUsage() plotGeneUsage()	V(D)J gene usage in general or stratified for functionality or junction frame usage (for subgroups, genes or alleles) + visualization method
compare.geneUsage() plotCompareGeneUsage()	Comparison of gene usage between different samples (for subgroups, genes or alleles) + visualization method
sequences.geneComb() plotGeneComb()	Gene/gene combinations for V(D)J genes (for subgroups, genes or alleles) + visualization method
aaDistribution() plotAADistribution()	Amino acid distribution of sequences of the same length + visualization method
compare.aaDistribution() plotCompareAADistribution()	Comparisons of amino acid distribution of sequences of the same length of different samples + visualization method
trueDiversity() plotTrueDiversity()	True diversity of sequences of the same length (Richness, Shannon, Simpson) + visualization method
compare.trueDiversity() plotCompareTrueDiversity()	Comparisons of diversity of sequences of the same length of different samples + visualization method
geneUsage.distance()	Several dissimilarity and distance measurements for gene usage data
sequences.distance()	Several dissimilarity and distance measurements for sequence data
dist.PCoA() plotDistPCoA()	Multidimensional scaling (principal coordinate analysis) of distances + visualization method

Parallel processing is possible for some methods using the *doParallel* package [12]. The number of computing cores is set by the user (single core processing by default). In S1 Table information about computational time and memory used for more complex functions is provided.

### Input data

The input data for *bcRep* are output tables of IMGT/HighV-QUEST. In total, IMGT/HighV-QUEST returns 10 tables (plus a parameter table and in some cases individual files). Tables required as input for the function are described in the corresponding help file. Functions to combine the output from several IMGT/HighV-QUEST output folders and to read in these tables are provided:

> combineIMGT(folders = c("pathTo/IMGT1a", "pathTo/IMGT1b", "pathTo/IMGT1c"),
name = "NewPro>ject”)
> readIMGT("PathTo/file.txt", filterNoResults = TRUE)

While reading input tables, sequences without any information (marked as “no results” in the “D-GENE and allele” column) can be excluded. IMGT/HighV-QUEST gives no results, when

1. The D gene and allele reference directory of the IGH analyzed sequences cannot be managed by the IMGT/GENE database.
2. Imprecise identification of the 3'V-REGION of the V gene and allele or/and of the 5'J-REGION of the J gene and allele.
3. The number of mutations in the V, D and/or J region is higher than a given threshold (set in preferences). [5]

### Sequence analysis

Functions to analyze features of the sequences from IMGT/HighV-QUEST output are implemented in the package. Information about functionality and junction frame distributions can be retrieved. Furthermore, filtering for subsets of functionality and junction frames is possible. Possibilities to analyze and visualize gene usage, as well as gene-gene combinations on subgroup, gene and allele level are given. For all these functions absolute or relative values can be returned.

**Fig 1:**
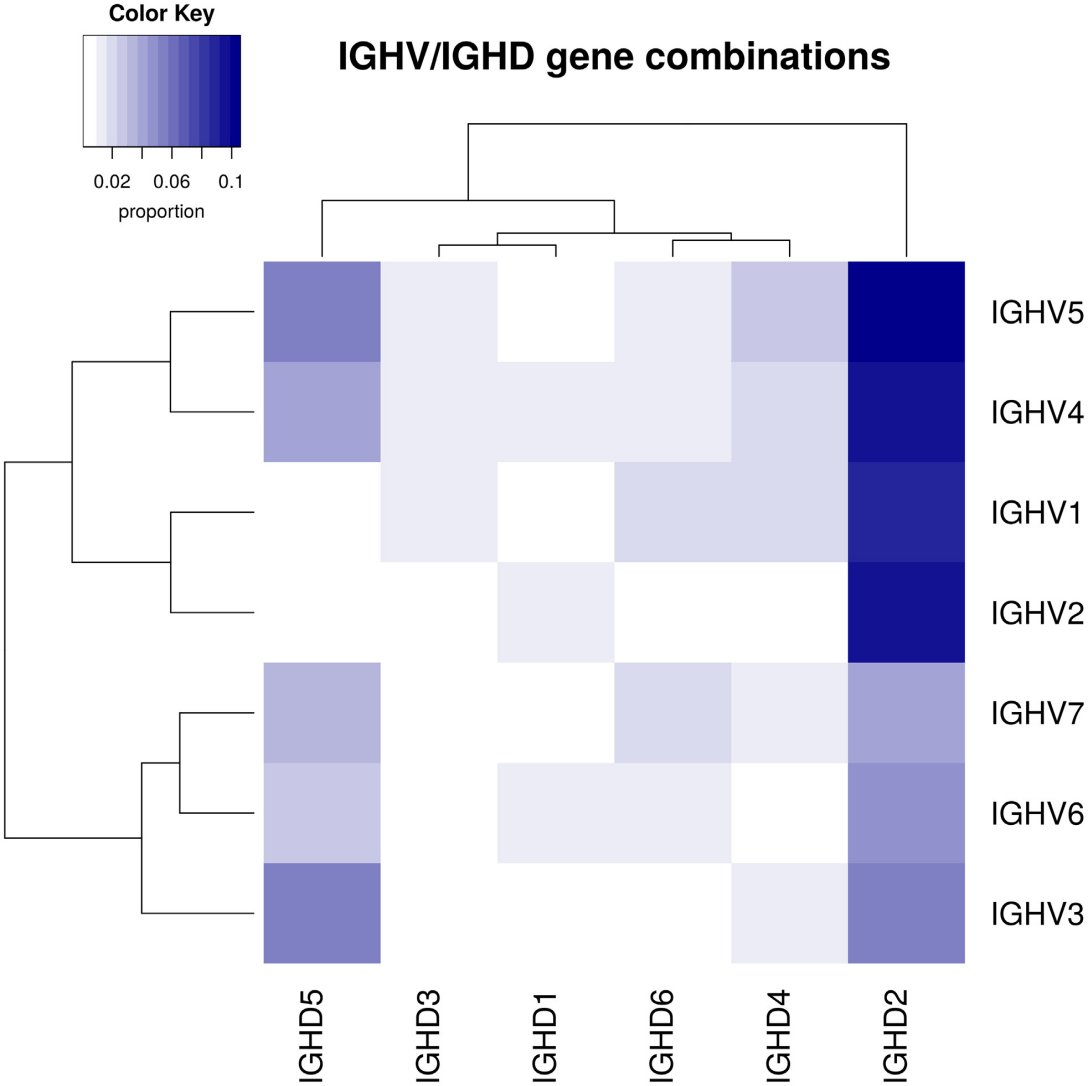
Example of an analysis of gene/gene combinations. A color coded heatmap represents the relative abundance of IGHV and IGHD combinations for a selected set of sequences. Bright colors represent low proportions, darker ones high proportions. Dendrograms represent hierarchical clustering of genes.

In Fig 1 an example of IGHV and IGHD gene combinations of a selected set of sequences is shown. Results are displayed as a heatmap, representing bright colors as low and darker ones as high proportions of gene/gene combinations. Further dendrograms are added to see how genes are clustered hierarchically. In the given example the most abundant combinations are IGHD2 combined with IGHV1, 2, 4 and 5. The corresponding functions are:

> sequences.geneComb(family1 = NULL, family2 = NULL, level = c("subgroup", "gene", "allele"), abundance = c("relative", "absolute"), nrCores = 1)
> plotGeneComb(geneComb.tab = NULL, color = c("gray97", "darkblue"), withNA = TRUE, title = NULL, PDF = NULL,…)

### Mutation analysis

Basic summary statistics about mutations, like R/S ratios (the ratio of replacement and silent mutations), are provided. IMGT/HighV-QUEST already provides tables containing general information about silent and replacement mutations, but no statistics. Silent mutations can be further analyzed by studying proportions of mutations from one to another nucleotide to find silent mutations that appear more often than others in a given set of sequences. Further methods to investigate nucleotide distributions of the environment of mutated positions. Therefore three positions up- and downstream of the mutated position are considered and ratios of mutation from one nucleotide to another are returned. This helps to get an overview about nucleotides that appear maybe more frequently at positions around the mutations.

**Fig 2:**
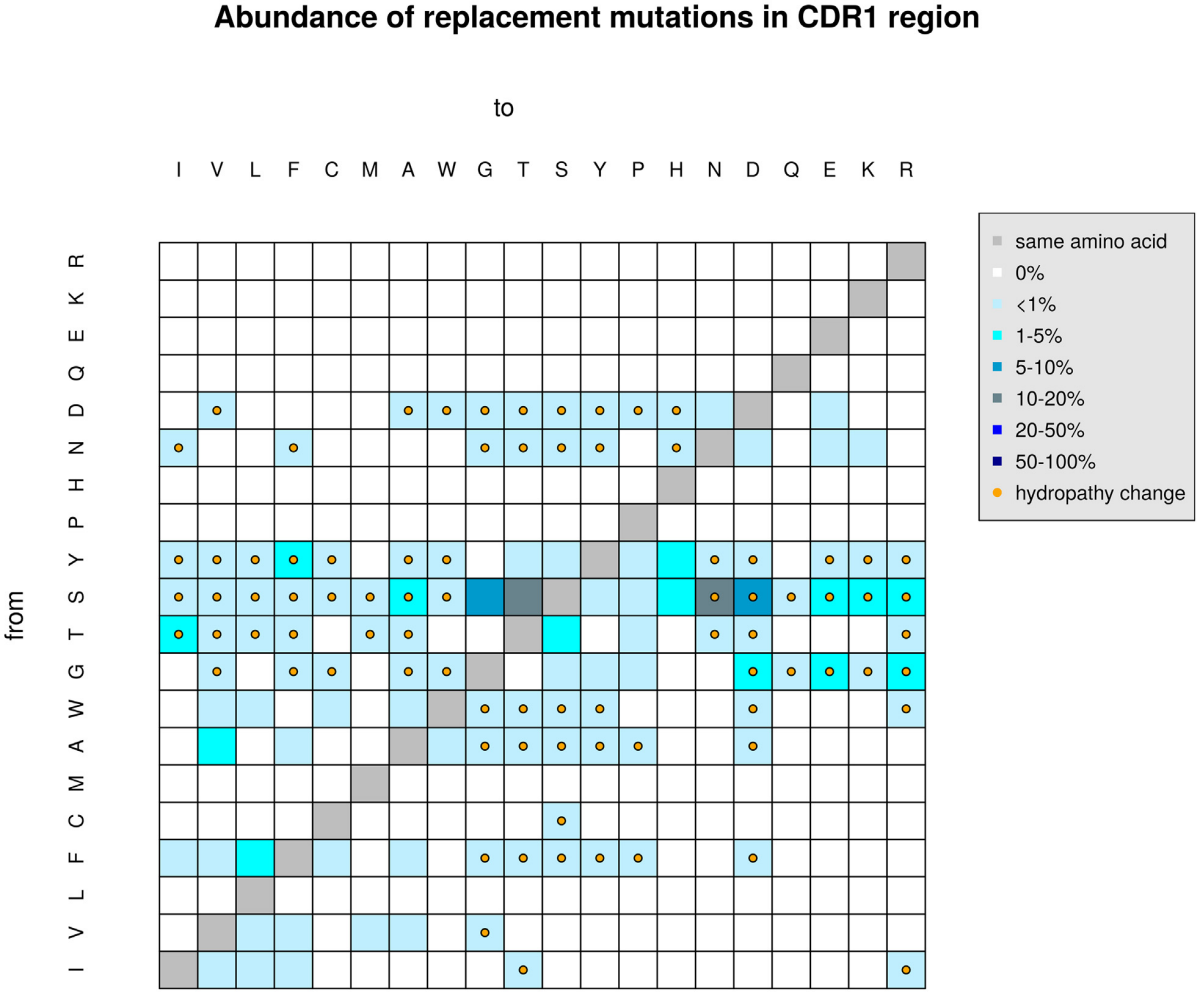
Example of an analysis of replacement mutations. Percentages of replacement mutations from one amino acid to another are color coded. Darker colors represent higher percentages, compared to bright colors. The amino acids of the germline sequence are shown in rows, the mutated ones in columns. The orange dots represent amino acid changes that result also in a hydropathy change.

Additionally, replacement mutations can be further analyzed. Here we concentrate on the appearance of certain mutations. Proportions of mutations resulting in amino acid replacements (reference amino acid according to germline identified by IMGT) are calculated to find substitutions that appear more often than others. In Fig 2 an example for the analysis of replacement mutations in CDR1 regions is provided. The percentages are color coded; darker colors represent higher percentages. Amino acids of the germline sequence are placed in rows the mutated ones are positioned in columns. Further replacement mutations resulting in hydropathy, chemical or volume changes can be highlighted. In the given example mutations from Serine (S) to Threonine (T) or Asparagine (N) appear most frequently (dark gray squares), but only the mutation from S to N imply also a hydropathy change (orange dots).

### Clone analysis

Clonotypes can be classified using different criteria regarding the complementary determining region 3 (CDR3), V and J genes. A threshold for CDR3 sequence identity can be chosen to either allow only identical CDR3 sequences (identity = 100%) or include possible somatic hypermutations (identity < 100%). It is mandatory to have the same V genes criterion. The application to same J genes is optionally. The user can select, how strong CDR3 identity shall be weighted and if sequences not only having same V genes, but also same J genes, shall be included. For instance iRAP considers same V, D and J genes and 100% CDR3 amino acid sequence identity. Change-O provides several methods to define clones: assigning total Ig sequences into clones, considering same V and J genes and a junction length with a specified substitution distance model or defining clones by specified distance metrics on CDR3 sequences and cutting of hierarchical clustering trees.

A function to look for clones shared between at least two samples is provided, as well. This function uses the same criteria as described above (clones). Additionally, a summary function is implemented. This function returns the number of clones per sample and the number of clones shared between different groups of samples.

Further clone features like copy number, CDR3 length, functionality, junction frames and gene usage can be analyzed and visualized. Filtering methods for clone size, functionality and junction frame usage are provided, as well.

**Fig 3:**
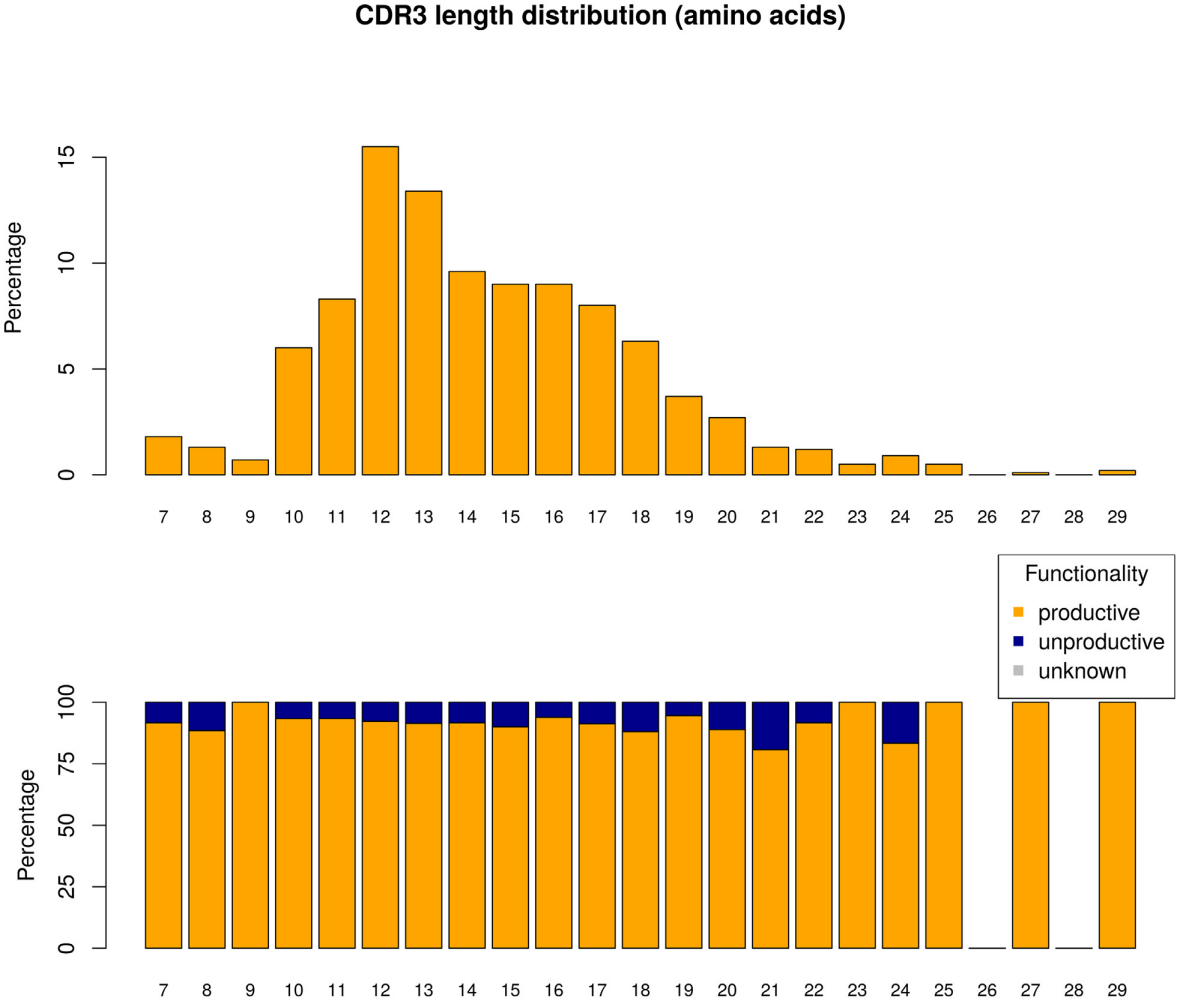
Example of an analysis of CDR3 amino acid sequence length distribution. a) Percentages (y-axis) of different CDR3 sequence lengths (x-axis) (upper figure). b) Percentages (y-axis) of productive (orange) and unproductive (blue) sequences per CDR3 sequence length (x-axis) (lower figure).

Functionality dependent of CDR3 length distribution can be visualized, using the function plotClonesCDR3Length() (Fig 3):

> plotClonesCDR3Length(CDR3Length = NULL, functionality = NULL, junctionFr = NULL,
color = c("orange", "darkblue", "gray"), abundance = c("relative", "absolute"),
title = NULL, PDF = NULL,…)

In the upper figure of Fig 3, the distribution of CDR3 lengths for a given set of sequences is shown. Most sequences have a length of 12 or 13 amino acids (each 13–15%). In the lower figure the percentages of productive and unproductive sequences (y-axis) dependent on the CDR3 length (x-axis) are displayed. Sequences with a CDR3 length of 21 or 24 amino acids have the highest percentages of unproductive sequences (> 12%).

### Diversity analysis

Functions for amino acid distributions, as well as diversity measurements are implemented.

A diversity index is a quantitative measure that reflects how many different types exist in a dataset. In our case types refer to amino acids per position. Simultaneously it takes into account how evenly the basic entities are distributed among those types. There are several diversity indices, which are simple transformations of the effective number of types, but each index can be interpreted as a measure corresponding to some real phenomenon.

The true diversity depends only on the value of sequence or amino acid frequencies and an exponent q, and not on the functional form of the index [13]. In almost all cases nonparametric diversity indices are monotonic functions of

Dq=(∑i=1npiq)1/(1−q),

or limits of such functions as q approaches unity. *D* is the effective number of types, *q* the order, *p*_*i*_ the relative abundance of species *i* and *n* the total number of species observed [13]. This means that when calculating the diversity of a set of sequences, it does not matter whether one uses Simpson concentration, inverse Simpson concentration or Shannon entropy; after conversion all give the same diversity. In Table 3 conversions of common diversity indices to true diversities are shown [13]. Diversities can be transformed in terms of the diversity index itself (*x*) or the proportions of the species (*p*_*i*_) [13].

**Table 3:** Conversion of specific diversity indices to true diversity indices [13].

Index x		Diversity in terms of x	Diversity in terms of p_i_
Species richness	$x=\;\overset{n}{\underset{i=1}{\sum }}p_{i}^{0}$	*x*	$\overset{n}{\underset{i=1}{\sum }}p_{i}^{0}$
Shannon entropy	$x=\;-\overset{n}{\underset{i=1}{\sum }}p_{i}\;ln\;\;p_{i}$	exp(*x)*	$\text{exp}\left(\;-\overset{n}{\underset{i\;=\;1}{\sum }}p_{i}\;ln\;p_{i}\right)$
Simpson concentration	$x\;=\;\overset{n}{\underset{i\;=\;1}{\sum }}p_{i}^{2}$	$\frac{1}{x}$	$\frac{1}{\sum_{i\;=\;1}^{n}p_{i}^{2}}$

The order of a diversity indicates its sensitivity to common and rare amino acids [13]. The diversity of order zero (q = 0) is completely insensitive to species (sequence or amino acid) frequencies and is better known as species richness [13]. Orders less than unity give diversities that disproportionately favor rare amino acids, while all values of q greater than unity disproportionately favor the most common species (sequences or amino acids) [13]. In the case of q = 1, all species are weighted by their frequency without favoring rare or common ones [13]. Regardless of q it always gives exactly n when applied to a community with n equally-common species.

True diversity (alpha diversity) can be analyzed using order zero (effective number of types (richness) [13]), one (Shannon entropy [14]) or two (inverse Simpson concentration [15]).

**Fig 4:**
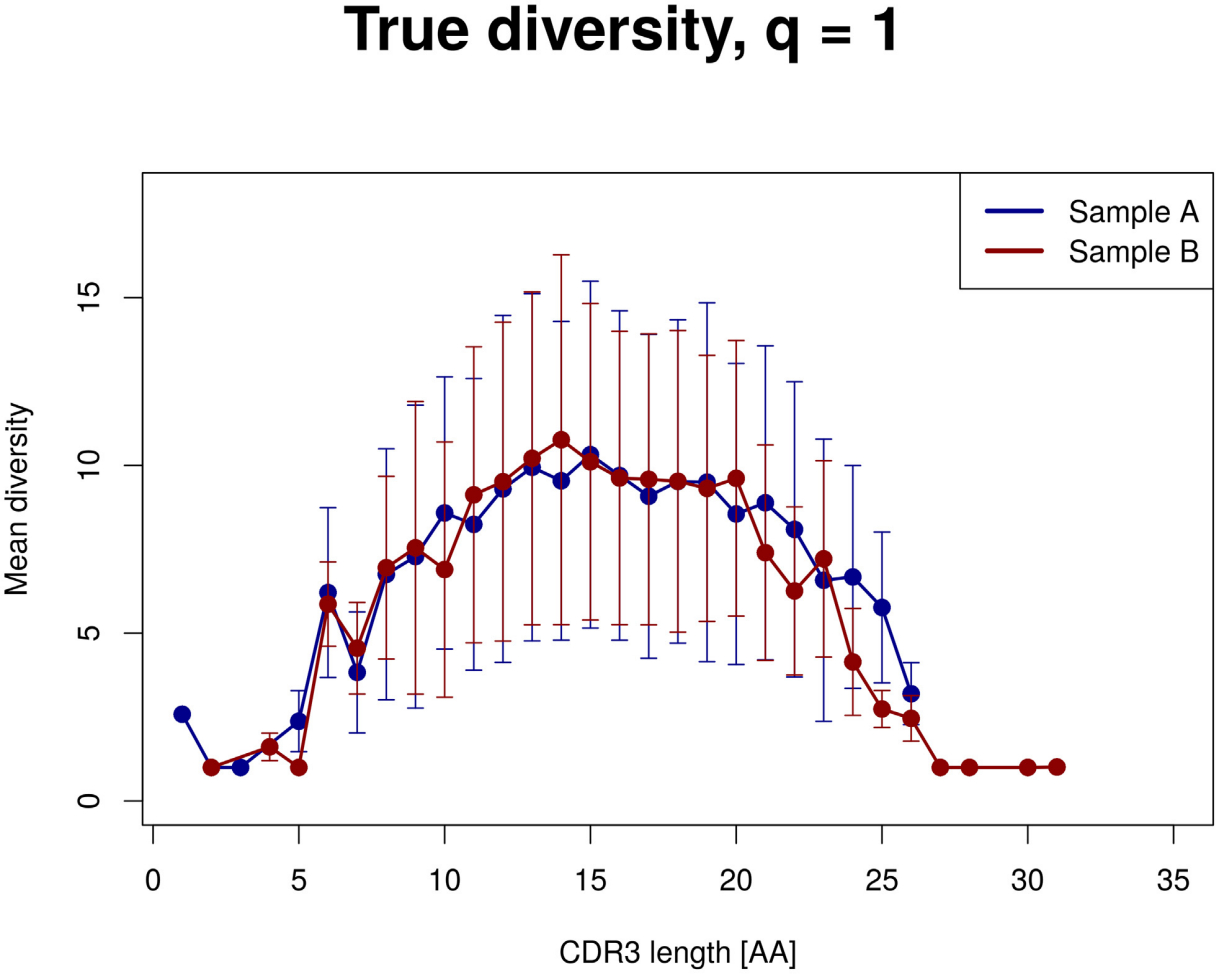
Example of a comparison of diversities of CDR3 sequences in two samples. Diversity indices of order one are given on the y-axis, CDR3 lengths (amino acids) are on the x-axis. Samples are color coded (blue and red). Dots represent mean diversities of all CDR3 sequences of given length; bars represent standard deviation. Diversity is alike in both samples, except for longer sequences (with a length of 21 to 26 amino acids), where CDR3’s of sample A are more diverse than those of sample B.

Diversity indices are calculated for sequences of the same length. Considering somatic hypermutations, deletions and insertions, it is difficult to assign CDR3 sequences to their native sequence and length. That is why diversity indices are calculated for each position. When visualizing the results, figures for each sequence length (x-axis: sequence position, y-axis: diversity index) or one figure including mean diversities and standard deviations (x-axis: sequence length; y-axis: mean diversity index) can be returned. An example is given in Fig 4, where mean diversity indices are compared between two samples (red and blue). Diversity is alike in both samples, except for longer sequences (with a length of 21 to 26 amino acids). For these positions CDR3 sequences of sample A are more diverse than of sample B. Also standard deviations differ for these sequence lengths. The corresponding functions for one or several samples are:

> trueDiversity (sequences = NULL, aaDistribution.tab = NULL, order = c(0, 1, 2))
> compare.trueDiversity (sequence.list = NULL, comp.aaDistribution.tab = NULL,
order = c(0, 1, 2), names = NULL, nrCores = 1)
> plotCompareTrueDiversity (comp.tab = NULL, mean.plot = T, colors = NULL, title = NULL, PDF = NULL)

Further a function calculating the Gini index, which measures the inequality of clone size distribution, is given. The Gini index is bound between zero and one. An index of zero represents a clone set of equally distributed clones, all having the same size whereas a Gini index of one would point to a set including only one clone. [16]

The corresponding function is:

> clones.giniIndex(clone.size = NULL, PDF = NULL)

**Fig 5:**
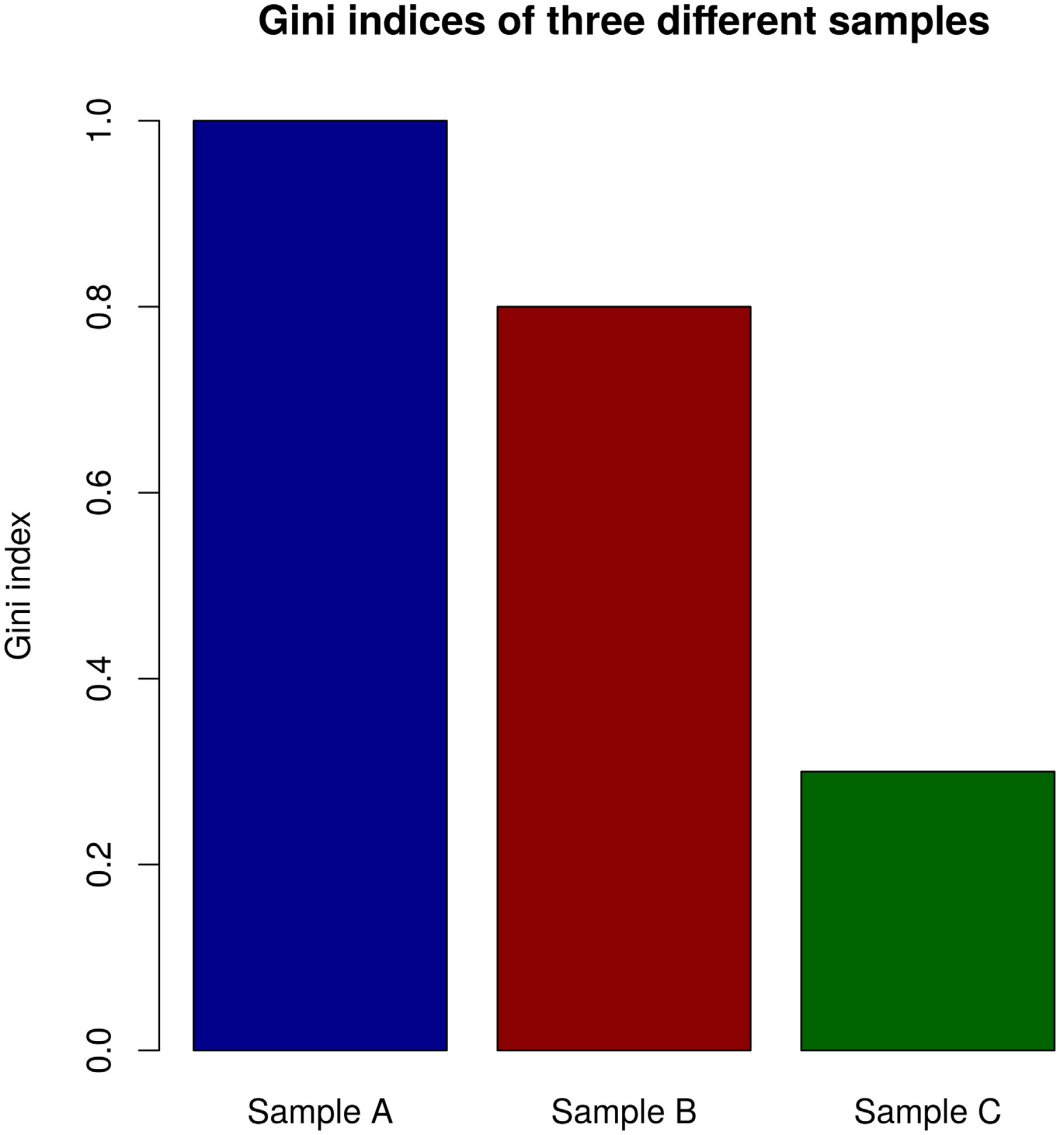
Example of a comparison of Gini indices of three samples. Gini indices are displayed on the y-axis, samples are on the x-axis. The Gini index can lie between zero and one. An index of zero represents a clone set of equally distributed clones, all having the same size. A Gini index of one would point to a set including only one clone with many sequences.

In Fig 5 an example of Gini indices for three different samples is given. Sample A has a Gini index of 1, which represents a set of only one clone including all sequences. Sample 2 is still dominated by big clones (with many sequences), but has also some clones with only few sequences (Gini index = 0.8). Sample 3 has a Gini index of 0.3, which means, that the clones are roughly equally distributed, but also some big clones exist.

### Comparison of different samples

There are some functions to compare data of different samples. For example, gene usage, amino acid distribution and diversity can be compared and results visualized across different samples. These functions need an input list containing sequence information from at least two individuals.

Additionally, clone sets of different samples can be compared. This function helps analyzing whether there are so called “public clones” that are shared among several samples or only “private clones” which represent each sample uniquely.

### Dissimilarity/distance measurements and multidimensional scaling

For gene usage, as well as for sequence data several dissimilarity and distance functions are provided. With these functions relationships between several samples can be analyzed (beta diversity). Dissimilarity, as well as distance measurements describes numerically how similar two objects are. For example, the Levenshtein distance [17], which represents the minimum of single-character edits between two sequences, would be two for the sequences “AABBCC” and “ABBBBC”, because there are two changes (second position A -> B, fifth position C -> B). Contrary, the longest common substring algorithm [18] returns an index of four (ABBC) for the given example. In the case of distances, higher values describe higher distances/dissimilarities. Small distances are equivalent to many similarities or little dissimilarity.

Studying distances between sequences can be done by either analyzing all input sequences together or analyzing subsets of sequences of the same length. Based on the R package *stringdist* [19] dissimilarity or distance indices like Levenshtein, cosine [20], q-gram [21], Jaccard [22], Jaro-Winker [23], Damerau-Levenshtein [24], Hamming [25], optimal string alignment [19] and longest common substring can be calculated. The indices are described more in detail in help files of *bcRep* and *stringdist* packages. For instance, Hamming distance only counts character substitutions between two sequences of the same length, whereas the Levenshtein distance also takes deletions and insertions into account. The optimal string alignment also allows for one transposition of adjacent characters, the full Damerau-Levenshtein distance allows for multiple substring edits. The q-gram, cosine, Jaccard and Jaro-Winkler distances underlie more complex algorithms.

For gene usage data a table containing gene proportions of different samples is required as input. When having samples in rows and genes in columns, the distances between the samples, based on the gene usage can be analyzed. Transforming this table will end up in distances between different genes, based on the different samples. Dissimilarity or distance measurements like Bray-Curtis [26], Jaccard or cosine are provided using implementations of the R packages *vegan* [27] and *proxy* [28]. Bray-Curtis is often used for abundance data, whereas Jaccard distance uses presence/absence data.

Further these results can be used to perform a multidimensional scaling (e.g. principal coordinate analysis, PCoA) and to visualize levels of similarity. Ordination methods, like PCoA can be used to display information contained in a distance matrix.

In the following example a distance matrix (cosine distance) is calculated, based on IGHV gene usage data of 42 samples. Afterwards PCoA is used to visualize the relationships between those samples. The 42 samples belong to two groups, for example a case and a control set.

> geneUsage.distance (geneUsage.tab = NULL, names = NULL, method = c("bc", "jaccard", "cosine"), cutoff = 0)
> dist.PCoA (dist.tab = NULL, correction = c("lingoes", "cailliez", "none"))
> plotDistPCoA (pcoa.tab = NULL, groups = NULL, names = NULL, axes = NULL,
plotCorrection = FALSE, title = NULL, plotLegend = FALSE, PDF = NULL)

**Fig 6:**
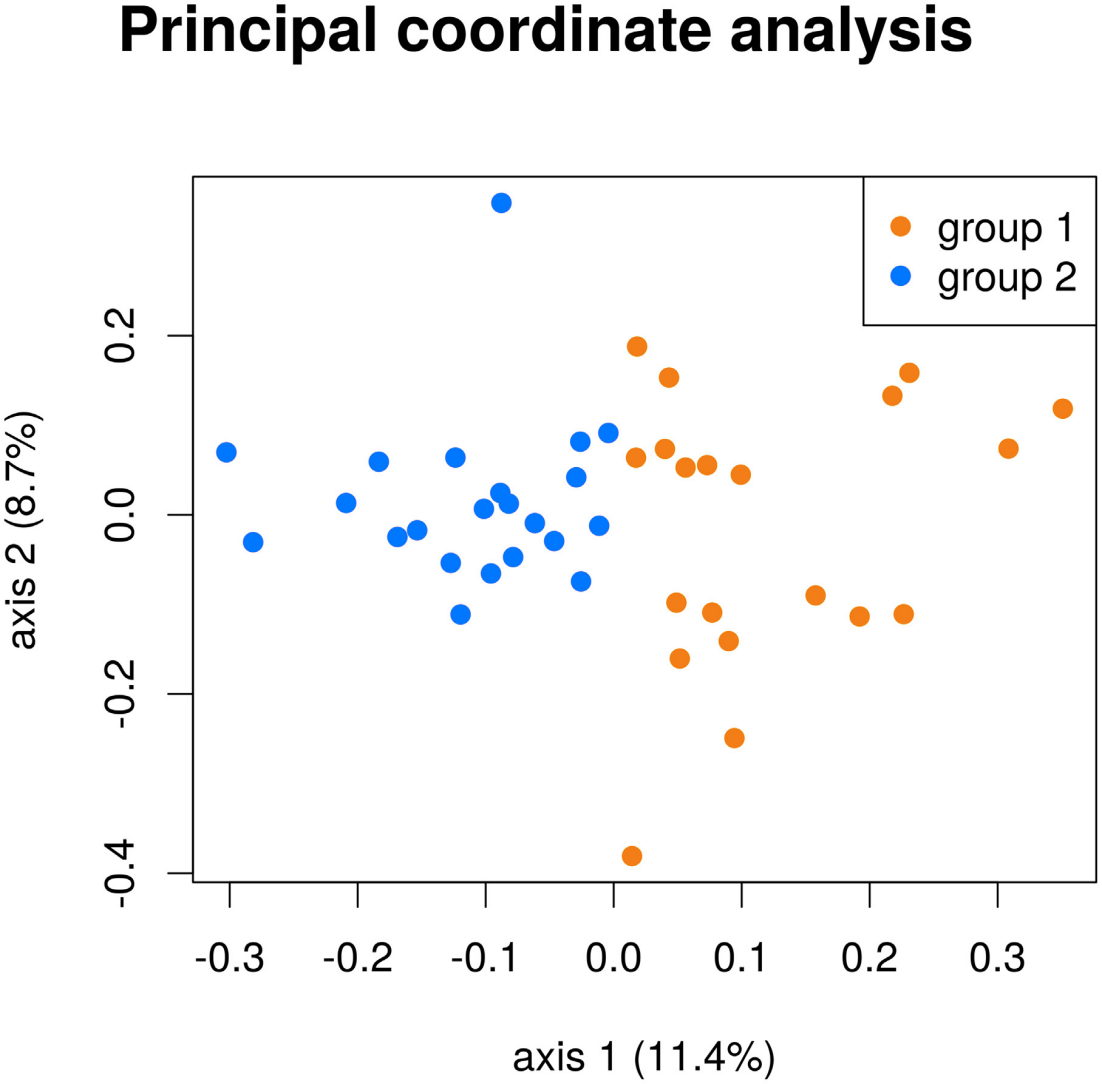
Example of a principal coordinate analysis based on cosine distances on IGHV gene usage distributions of 42 samples. The dots are color coded for two groups. First (x-axis) and second (y-axis) axes are plotted and the variances explained by these axes are given.

Fig 6 shows the first and second principal coordinate axes, explaining 11.4% and 8.7% of the total variance. Each dot represents a sample. Both groups are separated nicely (blue and orange dots). Further one can see, that group 1 is more diverse than group 2 (in group 2 the distances between the dots are less than for group 1). Finally one can assume that both groups underlie different IGHV gene usage distributions and the samples in group 2 are more similar to each other than in group 1.

## Conclusion

The *bcRep* package offers a new platform for comprehensive B cell receptor repertoire analysis. It combines several methods to summarize sequence characteristics of the underlying dataset in detail. Computation time can be reduced using parallel processing; however this is still dependent on the number of cores provided for analysis and the underlying computer architecture. *bcRep* can be used by scientists new to IG repertoire analysis, as well as by advanced users. Functions can be applied without reformatting the input data and most results can be visualized with implemented plotting routines included in this package. Advanced programmers can use the provided functions as entry for more thoughtful in depth analyzes.

A wide spectrum of methods analyzing individual samples, as well as comparing several samples is provided.

In future we plan to continue adding new methods of diversity analysis, clustering sequences into groups and comparing repertoires as well as methods for processing FASTQ or FASTA files.

## Supporting Information

S1 Table Computational time and object sizes of selected *bcRep* functions. Only more complex functions with high computational costs are chosen. Characteristics are shown for three samples with 1) only few sequences (Sample 1, n = 31 901 sequences), 2) a moderate number of sequences (Sample 2, n = 323 560 sequences)) and 3) many sequences (Sample 3, n = 928 225 sequences). Computational time is represented by CPU elapsed time (seconds) and memory by object size (Megabytes). For all functions only one core was used (no parallel processing). System features and selected parameters for functions are shown separately.
(PDF)
