# Supplementary material for: bcRep: R Package for Comprehensive Analysis of B Cell Receptor Repertoire Data
Source: PLoS One. 2016 Aug 23;11(8):e0161569. doi: 10.1371/journal.pone.0161569 (PMC4995022; doi:10.1371/journal.pone.0161569)
Supplement: S1 Table — Only more complex functions with high computational costs are chosen. Characteristics are shown for three samples with 1) only few sequences (Sample 1, n = 31 901 sequences), 2) a moderate number of sequences (Sample 2, n = 323 560 sequences)) and 3) many sequences (Sample 3, n = 928 225 sequences). Computational time is represented by CPU elapsed time (seconds) and memory by object size (Megabytes). For all functions only one core was used (no parallel processing). System features and selected parameters for functions are shown separately. (PDF) [file pone.0161569.s001.pdf]

S1 Table: Computational time and object sizes of selected *bcRep* functions. Only more complex functions with high computational costs are chosen. Characteristics are shown for three samples with 1) only few sequences (Sample 1, n=31 901 sequences), 2) a moderate number of sequences (Sample 2, n=323 560 sequences)) and 3) many sequences (Sample 3, n=928 225 sequences). Computational time is represented by CPU elapsed time (seconds) and memory by object size (Megabytes). For all functions only one core was used (no parallel processing). System features and selected parameters for functions are shown separately.

Processor	Intel Core i7-4770K CPU @ 3.50GHz x 8
OS type	64 bit
Memory	31.2 GiB
System	Ubuntu 14.04 LTS

	**Sample 1 (n=31901 sequences)**		**Sample 2 (n=323560 sequences)**		**Sample 3 (n=928225 sequences)**	
**function**	**CPU elapsed time (sec)**	**object size (MB)**	**CPU elapsed time (sec)**	**object size (MB)**	**CPU elapsed time (sec)**	**object size (MB)**
readIMGT()	1.20	24.52	9.93	194.30	26.08	707.46
clones()	26.66	2.39	120.45	37.31	1788.59	69.92
clones.giniInd()	0.009	0.000046	0.001	0.000046	0.014	0.000046
geneUsage()	1.05	0.013	12.34	0.01	36.51	0.014
sequences.mutation()	0.50	0.61	10.53	6.17	29.20	17.71
sequences.mutation.AA()	0.55	0.006	43.05	0.006	29.90	0.006
sequences.mutation.base()	3.34	0.004	169.88	0.004	125.48	0.004
sequences.functionality()	0.030	0.000893	0.33	0.000893	0.85	0.000893
trueDiversity()	1.84	0.11	10.76	0.11	46.63	0.12

**function**	**CPU elapsed time (sec)**	**object size (MB)**
clones.shared()	910.28	0.000038
compare.geneUsage()	221.89	0.009
geneUsage.distance()	0.002	0.000885
dist.PCoA()	0.008	0.003
compare.trueDiversity()	46.77	0.34

Parameters used in functions (default values were used for all other parameters):

- readIMGT(data = "1_Summary.txt", ...)
- clones(..., dispD = TRUE, dispCDR3aa = TRUE, dispCDR3nt = TRUE, ...)
- geneUsage(... , level = “gene”, ...)
- sequences.mutation(..., sequence = "CDR1", rsRatio = TRUE, ...)
- sequences.mutation.AA(..., sequence = "CDR1", ...)
- sequences.mutation.base(..., analyseEnvironment = TRUE, analyseMutation = TRUE, sequence = "CDR1", ...)
- trueDiversity(... , order = 1, ...)
- clones.shared(..., dispD = TRUE, dispCDR3aa = TRUE, dispCDR3nt = TRUE, ...)
- compare.geneUsage(..., level = "gene", ...)
- geneUsage.distance(..., method = "bc", ...)
- dist.PCoA(...)
- compare.trueDiversity(..., order = 1, ...)
